# Prevalence of enteropathogens and their antibiotic sensitivity pattern in puppies with hemorrhagic gastroenteritis

**DOI:** 10.14202/vetworld.2017.859-863

**Published:** 2017-08-04

**Authors:** A. Kokila Priya, M. Balagangatharathilagar, D. Chandrasekaran, M. Parthiban, S. Prathaban

**Affiliations:** 1 Department of Veterinary Clinical Medicine, Madras Veterinary College, Chennai - 600 007, Tamil Nadu, India; 2 Department of Clinics, Madras Veterinary College, Chennai - 600 007, Tamil Nadu, India; 3 Department of Animal Biotechnology, Madras Veterinary College, Chennai - 600 007, Tamil Nadu, India

**Keywords:** canine hemorrhagic gastroenteritis activity index, enteropathogens, fecal antibiotic sensitivity test, fecal polymerase chain reaction assay, hemorrhagic gastroenteritis

## Abstract

**Aim:**

Hemorrhagic gastroenteritis (HGE) ranging from mild to severe forms is commonly encountered in puppies. The aim of the study was to identify the prevalence of common enteropathogens and the antibiotic sensitivity pattern in puppies reported with HGE.

**Materials and Methods:**

The canine HGE activity index, with little modification, was adopted to identify Grade III/severely affected puppies below 6 months of age. Fecal polymerase chain reaction (PCR) assay was employed to screen and compare the enteropathogens in puppies with hemorrhagic diarrhea and healthy control.

**Results:**

Canine parvovirus 2b was identified in 90.3% of the diarrheic and 10% of the non-diarrheic healthy puppies. *Clostridium difficile* was identified in all the diarrheic puppies and in 80% of the healthy puppies. Among the diarrheic puppies, 17.7% were positive for *Clostridium perfringens* enterotoxin, 9.7% were positive for *C. perfringens* alpha toxin, 6.4% were positive for *Escherichia coli* shiga toxin, 6.4% were positive for *E. coli* enterotoxin (LT), and 3.2% were positive for canine distemper virus. Whereas, none of the healthy puppies were positive for these bacteria and toxins. Fecal antibiotic sensitivity test pattern revealed gentamicin to be sensitive in 95% of the cases, azithromycin in 50%, enrofloxacin in 25%, cefotaxime in 20%, and tetracycline in 5% of the cases.

**Conclusion:**

Parvoviral enteritis is predominant among puppies. Yet, bacteria and their toxins also play an important role in HGE. Gentamicin has higher sensitivity against the enteropathogens associated with the condition.

## Introduction

Acute hemorrhagic diarrhea is one of the most serious clinical manifestations of the gastrointestinal failure faced by small animal practitioners [1]. Infectious agents associated with diarrhea in young dogs are typically bacterial or viral [2]. The potential enteropathogenic bacteria associated with bacterial gastroenteritis in dogs include *Clostridium difficile, Clostridium perfringens, Escherichia coli, Campylobacter jejuni*, and *Salmonella* sp. [3]. While, the enteric viruses that are commonly detected in dogs with diarrhea include canine parvovirus (CPV), enteric *Canine coronavirus* (CCoV), and canine distemper virus (CDV) [4].

The intestinal micro flora of dogs and cats is a complex and poorly understood population. The poor understanding of what truly constitutes normal versus abnormal, along with the ability to only superficially characterize the gut microbial population, limits understanding of the pathophysiology of enteritis [5]. There is a growing body of evidence indicating bacterial translocation due to impaired gastrointestinal function, leading to the septicemia in young puppies [6]. This supports the use of antibiotics in the treatment protocol of the affected animals.

Thus, the study was conducted to identify the various possible underlying etiology of hemorrhagic gastroenteritis (HGE), which will help in better understanding of the pathogenesis and to identify the better choice of antibiotic in most of the affected puppies, thereby to improve the survivability.

## Materials and Methods

### Ethical approval

As the study was conducted with the clinical cases (affected animals) ethical committee approval was not required.

### Selection of animals and sampling

Puppies below 6 months of age presented to the Madras Veterinary College Teaching Hospital with hemorrhagic diarrhea were selected for the study. The selection criteria used were based on the canine HGE activity index [7] with little modifications, given in Table 1. A total of 62 puppies that satisfy Grade III/severely affected criteria were selected for the study. A total of 10 apparently healthy puppies below 6 months of age were selected as negative control. About 2 g of fecal sample and fecal swab was collected from the rectum of the affected puppies.

**Table-1:** Canine HGE index suggested by Unterer *et al.* [7] with little modifications.

Clinical signs	Grade 0	Grade I	Grade II	Grade III
Appetite	Normal	Mild	Moderate	Severely decreased
Vomiting frequency	No	1 X/day	2-3 X/day	>3 X/day
Stool consistency	Normal	Slightly soft	Very soft	Watery diarrhea
Stool frequency	Normal	2-3 X/day	4-5 X/day	>5 X/day
Dehydration	No	<5%	5-10%	>10%
Level of consciousness	Normal	Mild depression	Moderate depression	Severe depression/recumbency

HGE=Hemorrhagic gastroenteritis

Fecal samples collected from all the puppies during the study period were screened by polymerase chain reaction (PCR) for the common enteropathogens, including the bacteria, their toxins and viruses. The organisms that were screened include *E. coli* – shiga and enterotoxin (LT), *C. perfringens* – alpha (cpa) and *C. perfringens* enterotoxin (cpe) *C. difficile* and its toxin B, *Salmonella* sp., *Campylobacter* sp., and the viruses such as CPV-2b strain, CDV, enteric CCoV, and *Rotavirus*.

### Sample processing and DNA isolation (phenol chloroform method)

DNA isolation was performed using phenol-chloroform method [8]. 1-2 g of fresh fecal samples were collected. 3 ml of phosphate buffered saline was added to it. About 1 ml of this fecal mixture was taken, centrifuged at 6000 rpm for 10 min and the supernatant was collected. To 200 µL of supernatant, 800 µL of proteinase buffer, and 4 µL of proteinase K enzyme were added and incubated in a water bath at 37°C for 1 h. 800 µL of phenol:chloroform:isoamyl alcohol mixture (25:24:1) and 100 µL of 5 M sodium acetate were added and centrifuged at 10,000 rpm for 15 min. Then, 1 ml of isopropanol was added to the supernatant and kept at −20°C for 1 h or overnight incubation and centrifuged at 10,000 rpm for 15 min. 1 ml of 70% ethanol was added to the pellet and centrifuged at 10,000 rpm for 10 min. The ethanol was discarded and the pellet air dried. 30-50 µL of nuclease free water was added to the air-dried pellet and stored at −80°C until use.

### RNA isolation

RNA isolation from fecal sample was done using the TRIzol reagent method [9]. 250 µL of fecal sample (supernatant) was mixed with 750 µL of TRIzol reagent and incubated in ice for 2 min. 200 µL of chloroform was added, incubated for 5 min and centrifuged at 12,000 rpm for 15 min at 4°C. The 500 µL of isopropanol was added to the supernatant and kept at −20°C for overnight incubation. The samples were then centrifuged at 12,000 rpm for 20 min at 4°C. 1 ml of 70% ethanol was added to the pellet and centrifuged at 12,000 rpm for 15 min at 4°C. 15 µL of NFW was added to the RNA pellet. cDNA synthesis was performed with random primers and RevertAid M-MuLV reverse transcriptase as per the manufacturer’s instructions: The cDNA obtained was used as templates for PCR assay, the live attenuated Rotavirus vaccine (Human Rotavirus RIX 4414 strain) and killed CCoV vaccine (NL-18 strain) were used as positive controls.

### PCR

The stn gene corresponding to the *Salmonella* spp. was tested to show amplification with a molecular length of 617 bp [10]. The stx 1 (shiga toxin) and LT 1 (enterotoxin) genes corresponding to *E. coli* were tested to show amplification with a molecular length of 614 and 480 bp [11]. The 16S rRNA gene corresponding to *C. jejuni* and *C. coli* was tested to show amplification with a molecular length of 854 bp [12]. The 16S rRNA and toxin B genes corresponding to the *C. difficile* were tested to show amplification with a molecular length of 270 and 399 bp [13]. The cpa (alpha toxin) and cpe (enterotoxin) genes corresponding to the *Clostridium perfringens* were tested to show amplification with a molecular length of 400 and 233 bp [14]. The VP7 gene corresponding to *Rotavirus* was tested to show amplification with a molecular length of 1062 bp [15]. The VP2 gene corresponding to CPV-2b was tested to show amplification with a molecular length of 427 bp [16]. The H gene corresponding to CDV was tested to show amplification with a molecular length of 863 bp [17], and the S gene corresponding to CCoV was tested to show amplification with a molecular length of 346 bp [18], respectively.

The PCR reaction mixture containing 12.5 µL of master mix, 2 µL of DNA template and 6.5 µL of nuclease free water was taken in PCR tubes and kept on ice. The PCR tubes were spinned for 10 s and the appropriate program was set in thermal cycler.

### Agarose gel electrophoresis

The PCR products were analyzed in 0.8-1.5% agarose gel according to the PCR amplicon size, containing 1 µL of working ethidium bromide staining along with 100 bp DNA ladder [19]. The gel was visualized under ultraviolet transilluminator and documented using MEGA-CAPT software.

### Fecal antibiotic sensitivity tests (ABST)

Fecal ABST based on Kirby-Bauer agar disc diffusion technique was followed [20]. About 1-2 ml of nutrient broth was added to the culture swab and incubated at 37°C for 24 h. Mueller-Hinton agar plates were prepared, and the samples were inoculated. Antibiotic discs were placed at specific distance, and the plates were incubated at 37°C for 24 h. The results were read by measuring the zone of inhibition produced by various antibiotic discs and compared to the standards.

## Results

Based on the PCR assay, among the healthy puppies screened, 80% were found positive for *C. difficile*, and 10% were positive for CPV-2b.

**Figure-1:**
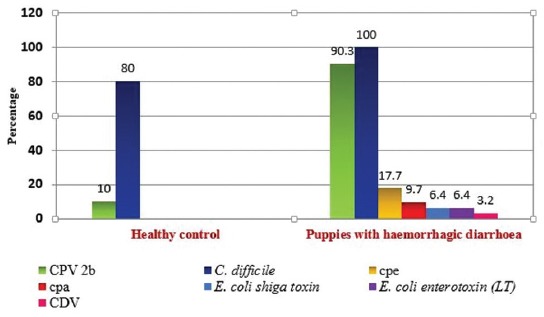
Enteropathogens identified in healthy and affected puppies.

Among the affected puppies, all were positive for *C. difficile*, 90.3% were positive for CPV-2b, 17.7% were positive for cpe, 9.7% were positive for cpa toxin, 6.4% were positive for *E. coli* shiga toxin (STEC), 6.4% were positive for *E. coli* – enterotoxin (LT), and 3.2% were positive for CDV (Figure-1). None of the puppies were shedding both the CPV and CDV together whereas, cpe together with cpa and LT were detected in two of the puppies with hemorrhagic diarrhea.

*Salmonella* sp.*, Campylobacter* sp., and *C. difficile* toxin B, enteric CCoV and *Rotavirus* were found negative in 62 puppies with hemorrhagic diarrhea screened samples.

The fecal antibiotic sensitivity results revealed gentamicin to be sensitive in 95% of the cases, azithromycin in 50%, enrofloxacin in 25%, cefotaxime in 20%, and tetracycline in 5% of the cases. The order of sensitivity was gentamicin > azithromycin > enrofloxacin > cefotaxime > tetracycline. Maximum resistance (100%) to amoxicillin and least resistance (5%) to gentamicin were observed.

## Discussion

The diagnostic methods based on PCR have the potential to be more sensitive and have a shorter turnover time, though they lack proper validation [21]. These molecular tests have been designed for the detection of many virulence genes and are often the most sensitive methods for detecting them [22].

PCR-based methods for CPV infection in dogs have been shown to be more sensitive than traditional techniques [23]. They have shown to detect very low concentrations CPV and feline parvovirus in unprocessed fecal samples, and have been more useful to diagnose and differentiate canine enteric pathogens as the definitive diagnosis is important primarily for epidemic control and prevention [24]. The PCR assays avoid the necessity for culture for subsequent phenotypic tests and that when employed to a large number of diarrheic samples help in clarifying the role of a particular organism in the enteric disease [12].

In this study, all the diarrheic puppies (100%) and majority of the healthy non-diarrheic puppies (80%) were positive for *C. difficile*. A variety of 20 species of bacteria and 10 species of fungi were isolated from the rectal swabs taken from healthy dogs [25]. Among them, *E. coli, Streptococcus mitis, S. lactis*, and *Enterococci* were more prevalent whereas *Clostridium* spp. and *Lactobacillus* spp. were least prevalent and neither *Salmonella* spp. nor *Shigella* were detected. Whereas, *Clostridium* spp. was most abundant in the fecal samples collected from dogs and cats (>20% on average) [26]. *C. difficile* was identified to cause of diarrhea in 10-21% of cases and postulated to be involved in some cases of acute hemorrhagic diarrhea syndrome in dogs [5,27]. Thus, the increased prevalence of *C. difficile* in the fecal samples in puppies is explained by their normal inhabitance and abundance in the gastrointestinal tract of the puppies, more commonly among the diarrheic animals.

The prevalence of CPV-2b in the diarrheic puppies (90.3%) was more when compared with the healthy non-diarrheic puppies (10%). The shedding of CPV and CDV is strongly associated with acute hemorrhagic diarrhea [4]. CPV-2 was typically seen in dogs without protective antibody titers, because of a lack of or an incomplete series of vaccinations [2]. A window of susceptibility also occurs in puppies, in which maternal antibody falls below protective levels but vaccine-induced immunity is lacking. CDV was involved in acute hemorrhagic diarrhea without prominent respiratory and neurological signs [28].

Cpe, cpa toxin, STEC, *E. coli* – enterotoxin (LT), and CDV were identified in the puppies with hemorrhagic diarrhea, but none of them were isolated from the healthy puppies.

*C. perfringens* was responsible for a wide range of diseases in humans and animals. The pathogenicity of this species is associated with the toxin production such as the major toxins- alpha, beta, epsilon and iota, and the minor toxin-enterotoxin (cpe) [29].

The identification of *C. perfringens* in the affected puppies could be related to the intestinal dysbiosis. The changes in the intestinal environment of dogs with diarrhea promote increased proliferation and transient overgrowth of enterotoxigenic strains of *C. perfringens*, leading to detectable amounts of enterotoxin (cpe) in the feces [30]. Frequent growth of *C. perfringens* was observed in the intestine of dogs with CPV infection [31].

The main pathogenic elements of STEC and enterotoxigenic *E. coli* were classified into shiga toxin (stx1, stx2), heat-labile toxin (LT), and heat-stable toxin (ST) [11]. A higher percentage of diarrheic dogs were positive for hemolytic *E. coli*, with increased prevalence in young age as the intestinal epithelium appears to be more permeable than is the intestinal epithelium in older dogs [32,33].

Amoxicillin-clavulanate was recommended in patients with bacterial translocation [34]. Gentamicin was identified as the drug of choice for treating Gram-negative gastroenteritis bacteria and parvoviral enteritis [35,36]. This supports the maximum sensitive pattern of gentamicin in 95% of the puppies with HGE. Cefotaxime was found useful against few Gram-positive and most of the Gram-negative microbes implicated in parvoviral enteritis [37], which was in turn found to be sensitive in 20% of the affected puppies. The choice and response to antibiotics vary with each and individual animal, as the type and composition of gastrointestinal microflora are not similar in all the animals [38] which explain the varying sensitivity pattern observed in the study.

## Conclusion

A higher prevalence of CPV-2b among the puppies with hemorrhagic diarrhea is evident in the locality. Effective vaccination program, client education, and disinfection strategies will help in reducing the incidence. *C. difficile* is identified to be a common enteropathogen in diarrheic as well as healthy puppies, yet its role in pathogenesis is not clearly understood. Various bacteria and the toxins also play an important role as a sole enteropathogen and in combination with viruses in the etiology of HGE in puppies. Gentamicin is found to have the maximum sensitivity pattern against the enteropathogens implicated in severe HGE.

## Authors’ Contributions

MB, DC, and MP helped to design the study. Sample collection and laboratory work was done by AKP. SP suggested necessary steps involved in the research throughout the study. All authors read and approved the final manuscript.

## References

[1] Dow S.W, Acute medical diseases of the small intestine (1996). Handbook of Small Animal Gastroenterology.

[2] Magne M.L (2006). Selected topics in pediatric gastroenterology. Vet. Clin. Small Anim.

[3] Marks S.L, Rankin S.C, Byrne B.A, Weese J.S (2011). Enter pathogenic bacteria in dogs and cats: Diagnosis, epidemiology, treatment and control. ACVIM Consens. Statement J. Vet. Intern. Med.

[4] Schulz B.S, Strauch C, Mueller R.S, Eichhorn W, Hartmann K (2008). Comparison of the prevalence of enteric viruses in healthy dogs and those with acute haemorrhagic diarrhoea by electron microscopy. J. Small Anim. Pract.

[5] Weese J.S, Staempfli H.R, Prescott J.F (2001). The roles of *C. difficile* and enter toxigenic *Clostridium perfringens* in diarrhoea in dogs. J. Vet. Intern. Med.

[6] Biffl W.L, Moore E.E, Role of the gut in multiple organ failure (2000). Textbook of Critical Care.

[7] Unterer S, Strohmeyer K, Kruse B.D, Sauter-Louis C, Hartmann K (2011). Treatment of aseptic dogs with haemorrhagic gastroenteritis with amoxicillin/clavulanic acid: A prospective blinded study. J. Vet. Intern. Med.

[8] Kumar M, Chidri S, Nandi S (2011). A sensitive method to detect canine parvo viral DNA in faecal samples by nested PCR. Indian J. Biotechnol.

[9] Budaszewski R.F, Pinto L.C, Weber M.N, Caldart E.T, Alves C.D.B, Martella V, Ikuta N, Lunge V.R, Canal C.W (2013). Genotyping of canine distempervirus strains circulating in Brazil from 2008-2012. Virus Res.

[10] Murugkar H.V, Rahman H, Dutta P.K (2003). Distribution of virulence genes in *Salmonella* serovars isolated from man and animals. Indian J. Med. Res.

[11] Osman K.M, Mustafa A.M, Elhariri M, Abd-Elhamed G.S (2012). Identification of serotypes and virulence markers of *Escherichia coli* isolated from human stool and urine samples in Egypt. Indian J. Vet. Microbiol.

[12] Linton D, Lawson A.J, Owen R.J, Stanley J (1997). PCR detection, identification to species level, and fingerprinting *Campylobacter jejuni* and *Campylobacter coli* direct from diarrheic samples. J. Clin. Microbiol.

[13] Struble A.L, Tang Y.J, Kass P.H, Gumerlock P.H, Madewell B.R, Silva J (1994). Fecal shedding of *Clostridium difficile* in dogs: A period prevalence survey in a veterinary medical teaching hospital. J. Vet. Diagn. Invest.

[14] Heikinheimo A, Korkeala H (2005). Multiplex PCR assay for toxin typing *Clostridium perfringens* isolates obtained from finish broiler chickens. Lett. Appl. Microbiol.

[15] Gouvea V, Glass R.I, Woods P, Taniguchi K, Clark H.F, Forrester B, Fang Z (1990). Polymerase chain reaction amplification and typing of *Rotavirus* nucleic acid from stool specimens. J. Clin. Microbiol.

[16] Park S.A, Park S.Y, Song C.S, Choi I.S, Kim H.Y, Lee J.B, Lee N.H (2012). Development of a novel vaccine against *Canine parvovirus* infection with a clinical isolate of the Type 2b strain. Clin. Exp. Vaccine Res.

[17] Aarthi K.S, Josewin S.W, Jojo N.E (2015). Phylogenetic analysis of fusion (F) and hemagglutinin (H) genes of canine distemper virus from field isolates in Tamil Nadu. Int. J. Pure Appl. Biosci.

[18] Pratelli A, Decaro N, Tinelli A, Martella V, Elia G, Tempesta M, Cirone F, Buonavoglia C (2004). Two genotypes of *Canine coronavirus* simultaneously detected in the faecal samples of dogs with diarrhoea. J. Clin. Microbiol.

[19] Minakshi S, Prasad G (2010). Rapid, sensitive and cost effective method for isolation of viral DNA from faecal samples of dogs. Vet. World.

[20] Reller L.B, Weinstein M, Jorgensen J.H, Ferraro M.J (2009). Antimicrobial susceptibility testing: A review of general principles and contemporary practices. Clin. Infect. Dis.

[21] Weese J.S (2011). Bacterial enteritis in dogs and cats: Diagnosis, therapy and zoonotic potential. Vet. Clin. Small Anim.

[22] Kilic A, Ertafi H.B, Muz A, Ozbey G, Kalender H (2007). Detection of the eaeA gene in *Escherichia coli* from chickens by PCR. Turk. J. Vet. Anim. Sci.

[23] Desario C, Decaro N, Campolo M (2005). Canine parvovirus infection: Which diagnostic test for virus?. J. Virol. Methods.

[24] Sakulwira K, Vanapontipagorn P, Theamboonlers A, Oraverakul K, Poovorawan Y (2003). Prevalence of *Canine coronavirus* and parvovirus infections in dogs with gastroenteritis in Thailand. Vet. Med. Czech.

[25] Clapper W.E, Meade G.H (1962). Normal flora of the nose, throat, and lower intestine of dogs. J. Bacteriol.

[26] Garcia-Mazcorro J.F, Lanerie D.J, Dowd S.E, Paddock C.G, Grützner N, Steiner J.M, Ivanek R, Suchodolski J.S (2011). Effect of a multi-species symbiotic formulation on fecal bacterial *Microbiota* of healthy cats and dogs as evaluated by pyrosequencing. FEMS Microbiol. Ecol.

[27] Cave N.J, Marks S.L, Kass P.H, Melli A.C, Brophy M.A (2002). Evaluation of routine diagnostic faecal panel for dogs with diarrhoea. J. Am. Vet. Med. Assoc.

[28] Decaro N, Camero M, Greco G, Zizzo N, Tinelli A, Campolo M, Pratelli A, Buonavoglia C (2004). Canine distemper and related diseases: Report of a severe outbreak in a kennel. N. Microbiol.

[29] Songer J.G (1996). Clostridial enteric diseases of domestic animals. Clin. Microbiol. Rev.

[30] Marks S.L, Kather E.J, Kass P.H, Melli A.C (2002). Genotypic and phenotypic characterisation of *Clostridium perfringens* and *Clostridium difficile* in diarrhoeic and healthy dogs. J. Vet. Intern. Med.

[31] Turk J, Fales N, Miller M, Paer L, Fesches J, Gasser H (1992). Enteric *clostridium perfringens* infections associated with parvo viral enteritis in dogs. J. Am. Vet. Med. Assoc.

[32] Ali D.H, Metwally A (2015). Characterization of enter pathogenic *E. coli* and antibiotic resistance properties in diarrheic pets. Alex. J. Vet. Sci.

[33] Munnicha A, Lubke-Becker A (2004). *Escherichia coli* infection in new born puppies. Clin. Epidemiol. Invest. Microbiol.

[34] Greene C.E, Schultz R.D, Greene C.E (2006). Immunoprophylaxis. Infectious Diseases of Dog and Cat.

[35] Banja B.K, Sahoo N, Das P.K, Ray S.K (2002). Clinic therapeutic aspects of gastroenteritis in dogs. Indian Vet. J.

[36] Ramprabhu R, Prathaban S, Nambi A.P, Dhanapalan P (2002). Haemorrhagic gastroenteritis in dogs - A clinical profile. Indian Vet. J.

[37] Reddy K.B, Shobhamani B, Sreedevi B, Prameela D.R, Reddy B.S (2015). *Canine parvo* viral infection in dogs and their treatment. Int. J. Vet. Sci.

[38] Suchodolski J.S, Simpson K (2013). Canine gastrointestinal microbiome in health and disease. Vet. Focus.

